# Cases Illustrative of the Influence of Belladonna

**Published:** 1861-04

**Authors:** Richard Hughes

**Affiliations:** Surgeon to the Brighton Orthopædic Hospital


*Cases Illustrative of the Influence of Belladonna.* By Richard Hughes, Esq., M. R. C. S., L. R. C. P., Ed., (Exam.) Surgeon to the Brighton Orthopædic Hospital.

[Dr. Hughes in two previous papers, one in the 'British Medical Journal' for June 2, 1860, and the other in the 'London Medical Review' for August, 1860, enters into the physiological effects of this medicine. He thinks that it is an *excitant of the sympathetic* and a *depressant of the cerebro spinal system.*]

In the following paper, says he, I propose to exhibit some results of the practical application of this theory, and to point out the further indications for practice thereby afforded. And I will begin with cases where the action of this drug upon the sympathetic system appears to be the *rationale* of its curative influence.

*Case* 1.—*Enuresis.*—July 1859. George M., aged 14, had long suffered from nocturnal incontinence of urine. There was nothing particular about the general appearance of the patient, or that of his urine. I ordered small doses of tincture of cantharides, which relieved him temporarily; but immediate relapse followed its discontinuance. I now prescribed one-third of a grain of extract of belladonna twice a day, in cannamon-water. In three days his complaint left him; a slight relapse, about a week subsequently, was immediately removed by a few more doses of the medicine; and he has since been entirely free of this ailment.

*Remarks.*—Incontinence of urine may obviously result from two immediate cases. 1. From over-action of the longitudinal fibres of the muscular coat of the bladder (detrusor urinæ.) This is usually excited by some irritating quality of the urine, and is best removed by alkaline medicines and opiate suppositories. 2. From weakness or paralysis of its sphincter. This second and far more common cause of enuresis, usually met with in delicate children, requires for its cure some agent capable of toning the sphincter muscle, either immediately, or through the medium of its nerves.—Such an agent, we have, according to my views in belladonna; and, accordingly, the accounts of the influence of this drug over the disease from all parts of Great Britian and the Continent are highly encouraging. It rarely fails to overcome it, and in its use is subject to none of the evils occasionally produced by cantharides.

In prescribing this drug for children, the discovery of Dr. Fuller, as to its comparatively slight effects on young subjects must be borne in mind; and what would seem to be large doses must be unhesitatingly used.

*Case* 2.—*Milk Abscess.*—July 1860, Emily H., aged 24, nine weeks after confinement caught cold. When I saw her, I found at the upper part of the left breast a hard swelling elevated above the surface, of about the size of a hen's egg; it was very painful, red, hot, and tender to the touch. There was also pain in the right side, constipation, headache, high fever; and in the course of the preceding night, there had been some delirium. The fever had somewhat abated since vomiting a quantity of pure bile early in the morning.—These symptoms pointed to concomitant derangement of the liver, and soon yielded to a mercurial pill and saline draught. The state of the breast I treated by suspending the mammæ by a handkerchief tied over the shoulder, and keeping the swelling covered with the unguentum belladonnæ of the *Pharmacopœia.* In twelve hours, all pain had ceased, and there had been no increase of swelling. Next day, the redness and heat had gone, some tenderness only remaining. The swelling remained stationary and slightly tender for a few days, then slowly disappeared. The infant was not put to the breast during the mother's illness, and the general fever was sufficient to diminish the secretion of milk; but, as soon as she became convalescent, the flow of milk in both breasts returned as free as before.

*Remarks.*—The localised inflammation of the breast to which (from its great tendency to suppuration) this term "milk abscess" is commonly applied, has now been frequently treated by belladonna. This drug has been ascertained to possess galactifuge properties; and accordingly, being applied in the form of extract or ointment around the nipple in these cases it speedily checks the secretion of milk, and with it the inflammation.

The galactifuge properties of belladonna are easily explicable upon the theory of its action which I have advanced. Cl. Bernard has found that there are two ways of diminishing secreting power of a gland—1, galvanising the sympathetic nerves which supply its bloodvessels; 2, dividing the cerebro-spinal nerves which supply its secreting cells. Belladonna, according to my view of it, both excites the sympathetic (as by galvanism), and depresses the cerebro-spinal influence (as by section) of the parts which it affects. The reresult of its application to a gland like the breast will therefore be to diminish alike its secreting power and the supply of the material on which that power is exercised. And this galactifuge property of the drug will be found invaluable in cases of still-birth, premature weaning, and others, where the check of secretion of milk is loudly called for.

But it will be noticed that in the case I have recorded, there was no check of the secretion of milk. Not to speak of the superior practical advantages of a plan of treatment which avoids this unpleasant concomitant, it is obvious that the *rationale* of the cure must be a different one. It seems to me to resolve itself into the simple action of belladonna in exciting the vaso-motor nerves, and thereby contracting the capillaries. The essence of the inflammatory condition, to my thinking, is a morbidly dilated state of these vessels. The exudation from them, on which Professor Hughes Bennet and his school lay so much stress, is surely a consequence only of their preternatural dilatation and fulness. A substance which will contract them, will save them the necessity of relieving themselves by exudation at all, and, even when this has taken place, will most surely promote its absorption. And this was the view of John Hunter. "If," he wrote in 1794, "we could discover an agent which would cause the contraction of the extreme vessels, we should have in this the true remedy for inflammation."

This case, then, of cure of the threatened milk-abscess by the local application of belladonna, appears to open a wide field for the treatment of inflammation in general. The three indications which inflammation gives us for treatment are—1. To moderate the force and frequency of the heart's action; 2. To diminish the superfibrination of the blood; 3. To reduce the hyperæmia of the part affected. The old mode of effecting the first two objects—in other words of relieving the symptomatic inflammatory fever—was to bleed, and to give mercury; which may fairly be described as robbing a patient of a portion of his life and poisoning the remainder. A more enlightened practice fulfills the former indication by antimony, aconite, and the other substances which have this physiological effect upon the heart; and the latter by low diet diluents, and salines. Third indication—the reduction of the hyperæmia of the parts—is commonly met by local bleeding and cold. But these are measures only applicable in their fullness to external inflammations, and even these are open to much improvement. Local bleeding, however efficacious, wastes blood, and leaves the capillaries weak and still morbidly dilated; and cold must be continuously applied, to be of avail. We should gain immensely, could we find a remedy capable of acting, whether locally or through the general system, upon the nerves of the inflamed part, so that its dilated capillaries might be aided to that contraction for which Nature is striving, and thus the hyperæmia of the part reduced without the loss of a drop of blood, or the inconveniences inseparable from leeches, cupping, and the continuous application of cold.

Such an agent I belive we have in belladonna; and I augur an immense advance in our control over inflammation as its use therein becomes extended within the limits which practical experience alone can define. (See the Lancet for August 4, 1860, for a case of myelitis treated by belladonna applied along the spine.)

*Case* 3.—*Congestive Headache.*—August, 1860. Mrs. M., aged 74, for four days in succession had been visited with severe headache, beginning with blindness, and lasting the greater part of the day. Similar headaches, some years ago, had been relieved by leeching. The administration of belladonna was commenced at the first coming on of the blindness, on the fifth day. The attack this day passed of sooner than usual. On the sixth day, there was but a slight attack; and since then there have been none.

*Remarks.*—The remarks made upon the last case will in most respects apply here, substituting congestion for inflammation. The congestive character of the headache seemed marked—1, by the accompanying blindness; 2, by the relief formerly afforded by leeches; 3, by the absence of good effect from a full dose of Hoffman's anodyne—a medicine which I find invaluable in cases of simple nervous headache. Here the belladonna accomplished the same good end as the leeches, without their expense, inconvenience, or spoliation of the vital fluid.

The result of this case would seem to indicate the use of belladonna to remove that habitual tendency to congestion of the brain which usually results in apoplexy.—*British Medical Journal.*

